# Interprofessional Education in Dentistry: Exploring the Current Status and Barriers in the United States and Canada

**DOI:** 10.7759/cureus.72768

**Published:** 2024-10-31

**Authors:** Abdul Khabeer, Muhammad Ali Faridi

**Affiliations:** 1 Undergraduate Medical Education, Schulich School of Medicine and Dentistry, University of Western Ontario, London, CAN; 2 Restorative Dental Sciences, College of Dentistry, Imam Abdulrahman Bin Faisal University, Dammam, SAU

**Keywords:** barriers to ipe, collaboration, curriculum integration, dental education, faculty engagement, healthcare teams

## Abstract

Interprofessional education (IPE) is defined as the collaborative learning process involving two or more healthcare professions to enhance teamwork and patient care. While the dental profession plays a key role in patient health, its integration into IPE remains underreported. This review examines the current status of IPE in undergraduate and graduate dental curricula, focusing on barriers such as time constraints, curriculum gaps, and limited faculty engagement. Through a review of the literature, including surveys and studies, findings suggest that while IPE has been increasingly incorporated into dental programs, challenges such as inadequate curricular integration and resource constraints hinder its full potential. Moreover, although dental students express positive perceptions toward IPE, translating these into actionable outcomes remains a challenge. The review also highlights the need for innovations, including online learning and faculty training, to overcome these barriers. Recommendations include integrating IPE into clinical training, improving faculty participation, and leveraging technology to foster collaboration and ultimately improve patient care outcomes.

## Introduction and background

The Centre for the Advancement of Interprofessional Education defines interprofessional education (IPE) as: “when two or more professions learn with, from, and about each other to improve collaboration and the quality of care” [1]. According to the Institute of Medicine (IOM), healthcare professionals should be trained to collaborate and communicate effectively in teams which would help in continuous and reliable patient care [2]. To achieve this goal, health professionals must be trained to work collaboratively either during their educational years or through continuous education. By incorporating IPE as a part of the educational curriculum, students from various health professions can learn about the roles and responsibilities of other professions. Therefore, dental schools and dental programs have become the focal point for the integration of IPE into the curriculum for dental students so that they can improve their interaction with different health professions from an early stage of their education [3].

Oral health plays a leading role in the well-being of an individual and dental professionals must be prepared to provide effective patient care. Findings suggest that there is an interaction between oral and systemic diseases with some oral diseases initiating certain systemic diseases, and vice versa [4]. This highlights the importance of IPE for dental professionals as they can act as the first line of defense by diagnosing and treating oral diseases, and potentially identifying other systemic diseases. In addition, with the help of IPE, dental and other health professionals can work collaboratively to treat oral or systemic diseases and provide effective patient care. This is supported by the “complexity theory” described by Sargeant (2009), which refers to system behavior that is dynamic and changing continuously [5]. Sargeant indicated that the focus should be shifted from learning individually in a specific profession and a multi-professional approach should be considered to meet the ever-changing needs of healthcare teams and systems. It has been shown that various healthcare professions are actively engaged in IPE; however, the involvement of the dental profession in IPE is still not well reported. As dental professionals are now expected to work collaboratively with other professionals, it is important to assess their learning needs, status, and barriers to IPE in this complex environment. Many dental professionals may not recognize the importance of IPE, face limited collaboration opportunities, and lack sufficient training in teamwork. Identifying and addressing these gaps is essential for improving their ability to work effectively with others and enhancing patient care and health outcomes. Therefore, this review aims to identify whether IPE is incorporated into undergraduate and graduate dental curriculums. Additionally, the review will examine if there are barriers to IPE in dentistry and what actions can be taken to improve its current status. This would be beneficial for dentists and other dental professionals who are in leadership positions and responsible for keeping the dental curriculum current. This paper will help identify the current involvement of dental professionals in IPE and what barriers exist to successfully implement IPE.

## Review

Methodology

**Figure 1:**
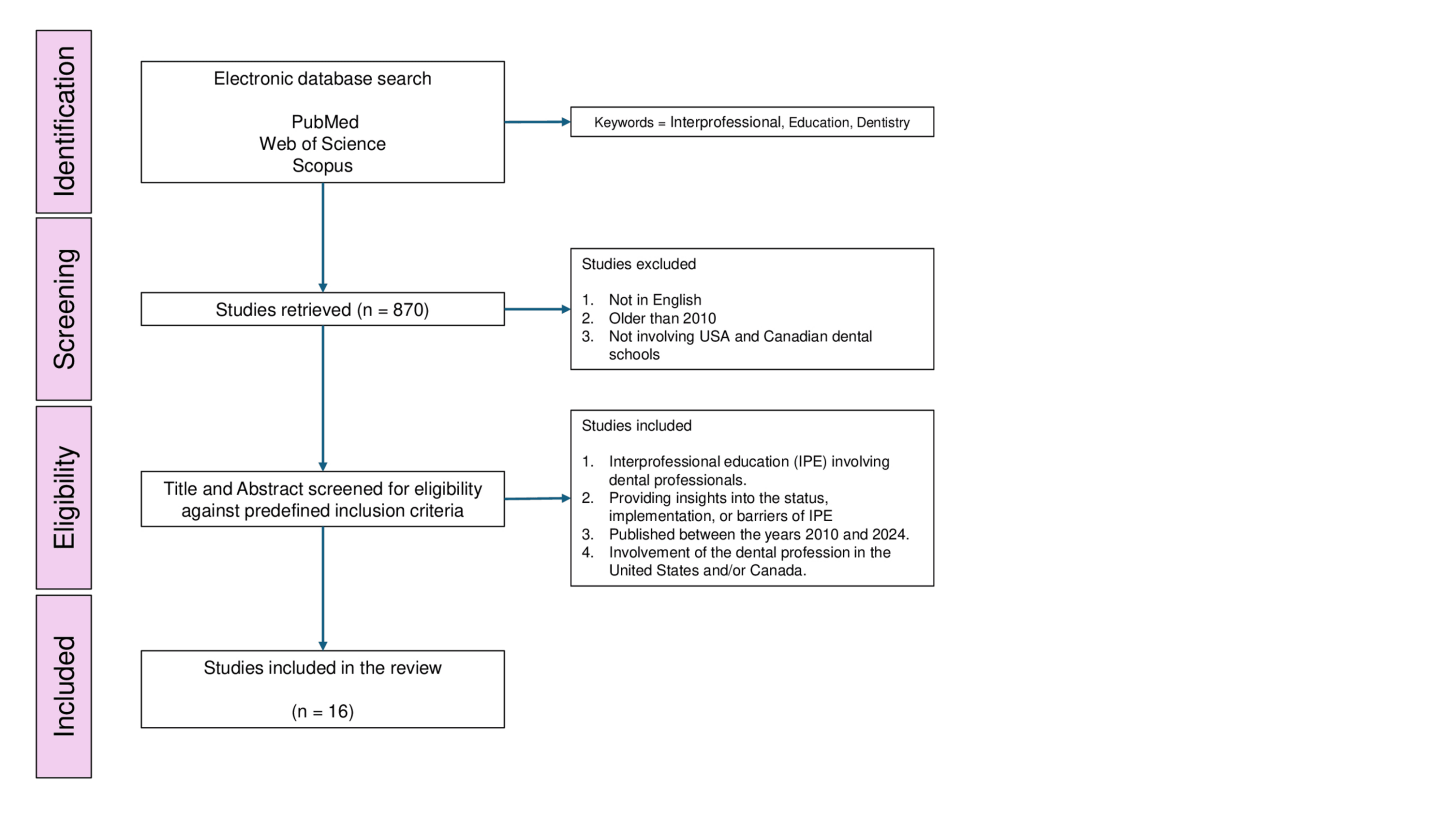
Flowchart showing the identification, screening, and selection of studies for this review.

A scoping review was conducted, beginning with an electronic search of the PubMed/MEDLINE (September 2024), Web of Science (September 2024), and Scopus (September 2024) databases. The search was carried out using the following criteria “interprofessional education”[MeSH Terms] OR (“interprofessional”[All Fields] AND “education”[All Fields]) OR “interprofessional education”[All Fields] AND ("dentistry”[MeSH Terms] OR “dentistry”[All Fields] OR “dentistry s”[All Fields]). A total of 870 articles were initially retrieved. After removing duplicates, titles and abstracts were screened against predefined inclusion and exclusion criteria. This review included studies that reported the dental professionals’ involvement in IPE; provided insights into the status, implementation, or barriers associated with IPE; published between 2010 and 2024; and focused on the dental schools of the United States and/or Canada. Exclusion criteria included articles not written in English, studies not focused on the United States or Canada, and those that did not directly pertain to dentistry or IPE. To ensure the reliability of the selection process, two independent reviewers assessed the articles, resolving any discrepancies through consensus. In the end, 16 journal articles were included in the study for review (Figure 1).

Status of IPE in Dentistry

Due to ever-changing demands, expectations, complexity, and health-related issues, there is an increased burden on healthcare professionals to work collaboratively. Similar to many health professions, the dental profession is an integral part of the healthcare system and dentists are expected to have effective communication skills to work collaboratively with other healthcare providers. With the help of collaboration, complex or chronic health issues such as diabetes can be managed more effectively as such diseases require a multidisciplinary approach between the dentist and other healthcare professionals [6]. Moreover, dental professionals are responsible for interacting with the public through community services to prevent dental-related diseases, such as dental caries, which can be achieved by engaging in IPE. It has been widely recognized that IPE plays a significant role in improving collaboration and teamwork among health professionals [7]. A survey conducted among graduating dental students in 2016 showed that students had a better understanding of patient care when interacting with other health professions [8].

An IOM report from 1995, Dental Education at the Crossroads: Challenges and Change, emphasized modernizing dental education by implementing an integrated dental curriculum [9]. This would mean that basic science subjects can provide clinically relevant knowledge, while the clinical subjects should include scientifically driven education. In addition, during the early 2000s, the American Dental Education Association (ADEA) emphasized the need for innovation in dental curricula by publishing a list of papers focusing on the need for change and innovation [10]. Both the IOM and ADEA suggested a multidisciplinary approach to support a unified goal of producing highly skilled and knowledgeable dental graduates. Traditionally, the dental curricula had been based on a heavy academic load that lacked clinical relevance. It has been reported by Hendricson (2012) that although the courses within the dental curriculum at one University in the United States were well organized, they lacked integration [11]. The learners are not taught to relate the information acquired in one course with another; therefore, disciplinary silos need to be taken down to promote curricular coherence.

A survey-based study was conducted by Haden et al. (2009) to determine the integration of IPE in dental schools within the United States and Canada [12]. They reported that only 43% of the responding schools gave students an opportunity to participate in other health professions education programs during the various stages of their education. Considering this low percentage of IPE involvement in dental programs, more emphasis needs to be placed on reforming the dental curriculum. Recently, a similar study was conducted by Elangovan et al. (2016) to report the updated status of IPE integration at dental schools in the United States and Canada [13]. It was shown that 90.3% of schools surveyed in the study reported integrating IPE into their curricula, with 58.1% of schools introducing IPE during the first and second years, while 48.4% and 45.2% of schools utilized IPE during the third and fourth years, respectively. Only two schools reported that some aspects of IPE were integrated during all years of their dental program. Although IPE has been implemented in the dental curriculum, a lack of integration still exists between the basic sciences and clinical courses [13]. This lack of integration highlights the importance of updating the dental curriculum, where the students can relate the theoretical knowledge attained during the basic sciences courses with the skills obtained during the clinical courses.

Recently, Jiang et al. (2020) reported the level of IPE involvement in dental schools in the United States [14]. A survey was sent to 66 dental schools to which 29 (43%) schools responded. Engagement in IPE was reported by 96% of the responding schools, with medical, pharmacy, and nursing students as the most commonly involved learners from other professions. Furthermore, dental schools responded that 79% of their faculty were involved in teaching at other health professional schools, while at the same time, 93% had faculty from other health professions teaching courses in the dental curriculum. Even though the majority of the responding schools were involved in IPE, very few were satisfied with the level of preparation and competence of students by graduation. Additionally, the specific curriculum years in which IPE was integrated were not reported.

Effectiveness of IPE

To determine the effectiveness of IPE, the outcomes need to be assessed; however, it can be challenging due to the heterogeneity of the methods used in various studies. Commonly, IPE learning has been assessed using the Readiness for Interprofessional Learning Scale (RIPLS) which is a pre and post-survey tool [15,16]. With the help of this tool, the perspectives of the students toward other health professionals and teamwork can be determined. A study conducted by Huebner et al. (2021) utilizing RIPLS reported the perception and attitude toward IPE in first-year health sciences students, including dental students, enrolled at a University in Canada [15]. They reported that students had a positive perception toward IPE with a score of 40.5/45 for domain 1 (teamwork and collaboration), 33.3/40 for domain 2 (professional identity), and 6.3/10 for domain 3 (roles and responsibilities). In addition, they reported that being a female, having a previous degree, or having past exposure to IPE were significantly related to positive IPE perception. Another study by Kersbergen et al. (2022) used RIPLS to report the change in the attitudes of graduate dental students related to IPE after working for one year in dental clinics [16]. They reported that although the dental students had a positive attitude at baseline, it did not improve further after attending the dental clinics. Price et al. (2024) in a survey of recently graduated dental students also revealed the positive feedback by the respondents with regards to IPE [17]. Even though the results of these studies are encouraging, further studies are required to determine if the perception of students can be translated into objective outcomes.

Barriers to IPE in dentistry

There are several barriers and facilitators to IPE at universities that have been reported in the literature for different health professions. These broadly include the participation of faculty members, time constraints, mode of teaching, location, curriculum, and resources. One of the approaches that have been taken is the development of the European Interprofessional Education Network Project, which attempts to overcome barriers and facilitate IPE by improving collaboration and better utilization of resources [18].

Participation

To achieve the desired learning outcomes of IPE, the students and faculty need to be motivated and should be open to changing their attitudes and ideologies about other professions. A study conducted by Reilly et al. (2014) assessed the changes in students’ knowledge and attitudes after participation in an interprofessional, team-based, geriatric home training program [19]. They reported that the fourth-year dental students showed a negative correlation between IPE and communication with patients and other professionals. This can be attributed to the traditional nature of the training received by dental students, whereby they are trained independently instead of a team approach utilized by other health professions. This highlights that changes need to be made in dentistry, and students and faculty members should be trained to promote collaboration, teamwork, and conflict resolution.

Time Constraints

One of the issues related to IPE is the availability of participants from different professions at a specific time. Due to the nature of the dental profession, scheduling issues can arise when implementing IPE activities. A study conducted by Jiang et al. (2020) reported that one of the major barriers to implementing IPE was related to the academic calendar, which was challenging for both private and public dental schools [14]. To overcome this barrier, online courses or modules can be utilized as they have shown to be effective [20]. A study conducted by Palumbo and Bennett (2016) reported that an online video conferencing approach for IPE involving students from eight disciplines, regarding caring for elderly patients with comorbidities in rural settings, resulted in a positive perception by all the participants [21].

Mode of Teaching and Location

One of the barriers to implementing IPE is the ease of access to the information or the location where IPE will occur. Traditionally, classroom settings or physical locations have been used where students or professionals from different disciplines come to participate. However, this can be demanding as it requires time commuting to and from the location which can have a negative impact on the participation of the learners. Recently, eLearning has been utilized to overcome this barrier and facilitate IPE. eLearning allows learners to participate from the comfort of their homes as it does not require expensive equipment. A study conducted by Edelbring et al. (2021) reported that students prefer to undertake IPE online rather than in person due to the flexibility allowed by online learning [22].

Curriculum

To achieve IPE, the curriculum must be updated with an emphasis on learning with other health professions. As suggested by the findings from a survey by Jiang et al. (2020), IPE has been incorporated at varying levels of dental school in the United States and Canada, this was further corroborated by de Mendonça et al. in a study conducted in 2024 [23]; however, Jian et al. also reported that the majority of these dental schools perceived the lack of time in the curricula as a major barrier to implementing IPE [14]. This highlights the challenge of dental students having a busy workload giving little opportunity for IPE. Therefore, efforts should be made to impellent IPE into existing clinical training where the students continue to learn from other professions with a unified goal of improving teamwork, communication, and collaboration. This would require that professional boundaries are taken down and each profession is welcoming of others, regardless of their title.

Resources

The availability of resources has shown to be a major barrier to implementing IPE. This would include, but is not limited to, the availability of time, space, staff, and finances. A study conducted by Fook et al. (2013) reported that a lack of resources such as the availability of staff and space is the most common barrier to successfully implementing IPE [24]. In addition, they also reported that the unavailability of faculty who are experts in the field can also have a negative impact on IPE. Moreover, Gilles et al. (2020) conducted a survey and reported that for the successful implementation of IPE, financial resources play a major role as there is a significant cost associated with designing and conducting IPE activities [25]. Therefore, the true potential of IPE needs to be realized by the stakeholders who are responsible for dedicating resources to such activities. One method of reducing resource constraints is a move to virtual learning, as highlighted by Chavis et al. in a study conducted in 2024 [26] without the need for extensive facilities and manpower [26]. Hence, further exploration of virtual methods of course delivery is needed.

Implications

The concept of IPE has gained popularity over the last decade as it has shown to play a major role in improving health professions education. It helps in promoting teamwork by enabling learners to learn about the perspectives of other healthcare professionals, allowing them to work collaboratively to meet the complex needs of their patients. By recognizing the roles and responsibilities of other professions, decision-making can be improved and ignorance within the team can be minimized. Despite the benefits, various barriers prevent the successful implementation of IPE. To overcome these barriers, the faculty needs to be trained in delivering IPE programs by experts in the field which will then allow the knowledge to be passed on to the students. Management should realize the importance of IPE and should promote positive change by setting expectations, allocating resources, and holding individuals responsible for the outcomes. Moreover, IPE should be incorporated into the existing curriculum and should be devised in collaboration with other healthcare professions which would allow for defining a unified goal for all the professions involved. Further, the scheduling of the IPE activities should be collaborated among various disciplines to incorporate IPE into the existing workload allowing them to learn with each other during their clinical training. Novel technologies should be utilized to deliver the IPE content, including online IPE activities, which would allow the learners to master the subject without placing the patient at risk. To achieve quality patient care, teamwork is essential, and IPE can play a vital link in achieving this goal.

Although rigorous search criteria were used, the findings could be improved by using a systematic review approach that follows established guidelines. This study only looked at schools in the United States and Canada, which limits the generalizability of the results. This narrow focus may miss important insights and practices from other countries. Future research should include a wider range of countries and educational settings, and consider using systematic review methods to strengthen the findings and make them more applicable.

## Conclusions

The future of dentistry holds promise, driven by changes in how students learn and practice. Dental professionals who have traditionally trained and worked individually are now encouraged to work in team-based settings due to growing demands and the evolving needs of their patients. This review highlights that IPE is a vital tool in health professions education to promote teamwork and collaboration. For this reason, IPE has been reported to be incorporated into the dental curricula at the majority of dental schools in the United States and Canada. Despite the growing recognition of IPE, its full potential in dental education remains under-researched. Barriers such as curriculum gaps, faculty engagement challenges, time constraints, and limited resources need to be addressed, requiring efforts at both the institutional and individual levels for better implementation. Addressing these barriers will require a multifaceted approach, including greater faculty involvement, integration of IPE into clinical training, and the use of innovative methods such as eLearning. Innovations and further studies are required to fully realize the true potential of IPE in dental education.
